# Wood-Derived Vascular Patches Loaded With Rapamycin Inhibit Neointimal Hyperplasia

**DOI:** 10.3389/fbioe.2022.933505

**Published:** 2022-07-19

**Authors:** Boao Xie, Liwei Zhang, Chunyang Lou, Shunbo Wei, Jing’an Li, Hualong Bai, Alan Dardik

**Affiliations:** ^1^ Department of Vascular and Endovascular Surgery, First Affiliated Hospital of Zhengzhou University, Zhengzhou, China; ^2^ School of Material Science and Engineering & Henan Key Laboratory of Advanced Magnesium Alloy & Key Laboratory of Materials Processing and Mold Technology (Ministry of Education), Zhengzhou University, Zhengzhou, China; ^3^ Key Vascular Physiology and Applied Research Laboratory of Zhengzhou City, Zhengzhou, China; ^4^ The Vascular Biology and Therapeutics Program, Yale University School of Medicine, New Haven, CT, United States; ^5^ Department of Surgery and of Cellular and Molecular Physiology, Yale University School of Medicine, New Haven, CT, United States

**Keywords:** wood, patch venoplasty, rapamycin, neointimal hyperplasia, vascular patch

## Abstract

**Background:** Patches are commonly used to close blood vessels after vascular surgery. Most currently used materials are either prosthetics or animal-derived; although natural materials, such as a leaf, can be used as a patch, healing of these natural materials is not optimal; rhodamine and rapamycin have been used to show that coating patches with drugs allow drug delivery to inhibit neointimal hyperplasia that may improve patch healing. Wood is abundant, and its stiffness can be reduced with processing; however, whether wood can be used as a vascular patch is not established. We hypothesized that wood can be used as a vascular patch and thus may serve as a novel plant-based biocompatible material.

**Method:** Male Sprague–Dawley rats (aged 6–8 weeks) were used as an inferior vena cava (IVC) patch venoplasty model. After softening, wood patches coated with rhodamine and rapamycin were implanted into the rat subcutaneous tissue, the abdominal cavity, or the IVC. Samples were explanted on day 14 for analysis.

**Result:** Wood patches became soft after processing. Patches showed biocompatibility after implantation into the subcutaneous tissue or the abdominal cavity. After implantation into the IVC, the patches retained mechanical strength. There was a significantly thinner neointima in wood patches coated with rapamycin than control patches (146.7 ± 15.32 μm vs. 524.7 ± 26.81 μm; *p* = 0.0001). There were CD34 and nestin-positive cells throughout the patch, and neointimal endothelial cells were Eph-B4 and COUP-TFII-positive. There was a significantly smaller number of PCNA and α-actin dual-positive cells in the neointima (*p* = 0.0003), peri-patch area (*p* = 0.0198), and adventitia (*p* = 0.0004) in wood patches coated with rapamycin than control patches. Piezo1 was expressed in the neointima and peri-patch area, and there were decreased CD68 and piezo1 dual-positive cells in wood patches coated with rapamycin compared to control patches.

**Conclusion:** Wood can be used as a novel biomaterial that can be implanted as a vascular patch and also serve as a scaffold for drug delivery. Plant-derived materials may be an alternative to prosthetics or animal-based materials in vascular applications.

## Introduction

The application of plant-based scaffolds to treat human patients breaks the boundaries of plants and animals. Traditional animal-based scaffolds have several issues with use in human patients, including the need for decellularization before use to prevent immune reaction and rejection, the potential for transmission of zoonotic diseases, and religious and ethical objections to use in some people. Plant-based scaffolds avoid these issues and have been tested in cell culture with promising results for several applications. For example, vegetables such as spinach and parsley can be used in endothelial cell and stem cell culture; these plant scaffolds can be recellularized with human cells that adhere differentially to various surfaces of the scaffolds; decellularized apple, carrot, and celery maintained their porous structure and support cell adhesion, proliferation, and differentiation (Gershlak et al., 2017), (Contessi Negrini et al., 2020). Plant-derived scaffolds can be used as a platform to create vascular patches and drug delivery scaffolds. In particular, decellularized leaf scaffolds can be loaded with polylactic-co-glycolic acid (PLGA)-based rapamycin nanoparticles to inhibit venous neointimal hyperplasia in a rat inferior vena cava (IVC) venoplasty model; decellularized onion cellulose patches can also be coated with PLGA-rapamycin nanoparticles to inhibit venous neointimal hyperplasia (Bai et al., 2021a). Furthermore, it is possible to capitalize on the natural absorption capability of fresh leaves; fresh leaves absorbed with rapamycin and the IL-33 antibody can effectively inhibit venous neointimal hyperplasia (Xie et al., 2021). These data show promising potential applications of plant-derived biomedical materials.

Leaves are water-resistant and have a thick, astomatous cuticle on their surface, composed of cutin and waxes (Petit et al., 2021). The cuticle inhibits cell migration into the leaf, preventing host cell replacement (Xie et al., 2021). Modulevsky et al. (2016) created implantable cellulose scaffolds from apples that were subcutaneously implanted in mice and showed biocompatibility; after implantation, cells migrated into the spaces of the cellulose, and angiogenesis occurred within the cellulose scaffold. Although these plant materials showed interesting and exciting results, wood has never been tested in animal experiments. Wood is an abundant material and is widely used in many applications; wood-based scaffolds contain a naturally aligned microchannel structure, potentially facilitating biological applications. Although wood is hard and stable in shape, with very low compliance that requires treatment for applications, different trees produce wood with different characteristics that may allow processing steps that achieve material properties that are compatible with human use.

Recently, one group has reported a novel processing strategy that uses cell wall engineering to shape flat sheets of hardwood into versatile three-dimensional (3D) structures, allowing the material to be folded and molded into desired shapes (Xiao et al., 2021). This modified wood showed promising application, but whether wood can be used as a vascular patch and drug delivery scaffold is unknown. We hypothesized that modified wood can be used as a vascular patch and can also be loaded with rapamycin to inhibit neointimal hyperplasia in a rat patch venoplasty model. Here, we implanted processed wood patches in the subcutaneous and abdominal cavity, as well as into blood vessels; the capability for drug delivery was tested using rhodamine and rapamycin.

## Methods

### Fabrication Process of the Moldable Wood

**FIGURE 1:**
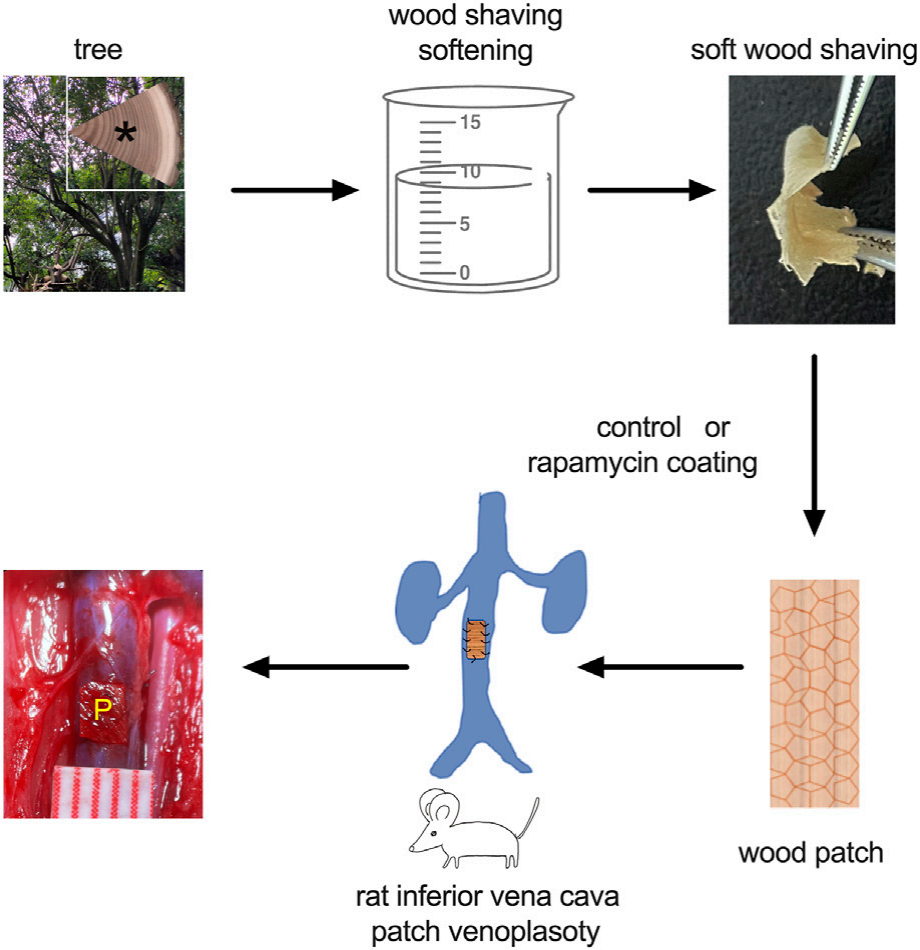
Illustration of the study design. * shows the area from which the wood was harvested.

The procedure to fabricate the wood patch was carried out as previously described (Xiao et al., 2021). Na_2_SO_3_ (>98%) and NaOH (>98%) were used for delignification; since lignin contributes to the stiffness and rigidity of wood, delignification is needed to decrease the stiffness of the wood patch prior to implantation. First, natural willow wood pieces (0.3 mm × 10 cm x 10 cm) from the tree trunk (Figure 1) were boiled in a solution of 2.5 M NaOH and 0.4 M Na_2_SO_3_ for 8 h, rinsed completely to remove the chemicals, dried at room temperature for 16 h, and then immersed in water for 3 min to form the moldable wood. Finally, the samples were air-dried at room temperature for 6 h and stored at −20°C for later use.

### Coating of the Wood Patch

Patches were coated with hyaluronic acid (HA) and either rhodamine or rapamycin; coating a patch with HA is an effective and commonly used method to deliver therapeutic drugs. HA can be combined with a drug *via* chemical coupling and coated onto the surface of a patch, which not only enables the patch to release the drug but also increases patch biocompatibility. The fluorescence of rhodamine released from the graft can be used to determine the drug delivery capability of the graft.

The procedure to coat the patches was carried out, as previously described (Bai et al., 2021b; Bai et al., 2022a). Briefly, the wood was immersed in a HA solution (2 mg/ml; molecular weight of 100,000 Da; Bloomage Biotech, China) for 15 min. The HA solution was activated in advance in a water-soluble carbodiimide solution and incubated for 6 h at 37°C, after washing three times with phosphate-buffered saline (PBS; 5 min/wash). The HA-coated samples were immersed into a rapamycin solution (2 mg/ml; Zhaoke, Hefei, China) that was also advance-activated in a water-soluble carbodiimide solution (15 min) and incubated at 37°C for 6 h. Rhodamine (Aladdin, 81-88-9; Shanghai, China) conjugation was carried out in a similar fashion.

The morphology and roughness of delignified uncoated and HA-coated patches were observed using a 3D optical microscope (NPFLEX, Bruker, Madison, WI, United States), with different surface colors used to represent different roughness and morphologies. (Wang et al., 2020).

### Animal Model

The study was approved by the First Affiliated Hospital of Zhengzhou University, Animal Care and Use Committee. All animal care complied with the Guide for the Care and Use of Laboratory Animals. NIH guidelines for the care and use of laboratory animals (NIH Publication #85–23 Rev. 1985) were observed. Male Sprague–Dawley (SD) rats (6–8-week-old) were used for all the animal experiments. Anesthesia was administered by intraperitoneal (IP) injection using 10% chloral hydrate (0.2–0.3 ml/100 g).

The inferior vena cava (IVC) patch angioplasty models were performed, as previously described (Bai et al., 2017a; Bai et al., 2021c). Briefly, the rat abdominal IVC was exposed and dissected free of surrounding structures, and the IVC was clamped; a longitudinal 3 mm venotomy was then made on the anterior IVC wall; a control wood (without coating), rhodamine-coated wood patch, or rapamycin-coated wood patch (3 × 2 mm) was sutured in place using running 10-0 nylon sutures. After completion of venoplasty, the microclamps were removed, and the IVC flow was restored. The abdomen was then closed using 5–0 Dacron sutures. Rats were sacrificed on postoperative day 14, and the patches were explanted for analysis. To avoid confounding or off-target effects, no immunosuppressive agents, antibiotics, antiplatelet agents, or heparin were given at any time during or after the operative procedure.

For the subcutaneous and abdominal cavity implantation models, the control or rapamycin-coated wood patches were implanted subcutaneously and into the abdominal cavity. Patches were harvested on day 14 for analysis. Rhodamine-coated wood patches were implanted subcutaneously and harvested on day 14.

On day 14, the rat was anesthetized, the chest was opened, the left ventricle was cannulated with a blunted 20-gauge needle connected to a 20-ml syringe, and an incision was made in the right atrium to allow outflow of perfusion solutions; 100 ml of phosphate-buffered saline (PBS) was infused, followed by 10% formalin being perfused by manual pulsatile syringe pressure. The patch or the tube graft was carefully removed from the surrounding tissue and stored in 4% neutral buffered formaldehyde.

### Histology

Rats were anesthetized with 10% chloral hydrate (IP injection), and tissues were fixed by transcardial perfusion of phosphate-buffered saline (PBS), followed by 10% formalin. Tissues were removed and fixed overnight in 10% formalin, followed by 24-h immersion in 70% alcohol. Tissues were then embedded in paraffin and sectioned (4 μm thickness). Tissue sections were deparaffinized and stained with hematoxylin and eosin (Baso, Zhuhai, China), according to the manufacturer’s recommendations. Neointimal and adventitial thickness were measured as previously described (Bai et al., 2021a). The patency of the patch angioplasty was confirmed by observation of the histology sections.

### Immunohistochemistry

Sections were heated in citric acid buffer (pH 6.0, Beyotime, Shanghai, China) at 100°C for 10 min for antigen retrieval. Sections were then treated with 0.3% hydrogen peroxide for 30 min and incubated overnight at 4°C with primary antibodies. After overnight incubation, the sections were incubated with appropriate secondary antibodies for 1 h at room temperature and treated with a 3,3N-diaminobenzidine tetrahydrochloride horseradish peroxidase Color Development Kit (Beyotime, Shanghai, China) to detect the reaction products. Finally, the sections were counterstained with hematoxylin (Baso, Zhuhai, China).

### Suture Retention

Suture retention of the decellularized patches was measured (Bai et al., 2021b). Briefly, suture retention testing was performed on rectangular specimens (2 cm × 1 cm) clamped at the edges and located opposite to an 8-0 Prolene suture anchored 5 mm from the edge; the suture loop was pulled with a tension meter, and when the suture tore through the patch, the tension was recorded.

### Immunofluorescence

Tissue sections were deparaffinized and then incubated with primary antibodies overnight at 4°C. The sections were incubated with secondary antibodies for 1 h at room temperature, after which sections were stained with DAPI (Solarbio, Beijing, China) to mark cellular nuclei. Rhodamine-conjugated patches were processed as mentioned previously and observed directly under the fluorescence microscope.

### Saffron-O and Fast Green Staining

Tissue sections were deparaffinized with water, stained by Saffron-O staining (Solarbio, Beijing, China) for 1 h, and then washed with tap water to remove the excess dye; the slides were again treated with 50, 70, and 80% gradient alcohol for 1 min, and were stained with the Fast Green Staining Solution (Solarbio, Beijing, China) for 1 min; the slides were dehydrated in absolute ethanol twice and made transparent with xylene.

### Antibodies

Primary antibodies included the following: anti-CD68 (Abcam, ab31360; IF, 1:50); anti-CD31 (R&D, AF3628; IHC, 1:100); anti-α-actin (Abcam, ab5694; IF, 1:200); anti-CD34 (Abcam, ab81289; IF, 1:50); anti-COUP TF II (Abclone, A10251; IHC, 1:50); anti-Eph-B4 (Proteintech, 20883-1-AP; IHC, 1:50); anti-nestin (Abcam, AB11306; IF, 1:50); anti-PCNA (Abcam, ab29; IF, 1:100); anti-piezo1 (ABclonal, A0659; IF, 1:100); and vWF (von Willebrand Factor, Abcam, ab11713, 1:100). Secondary antibodies used for IF were from ABclonal, Wuhan, China.

### Statistics and Reproducibility

Data are expressed as the mean ± SEM. Statistical significance for these analyses was determined by the t-tests (Prism 6; GraphPad Software, La Jolla, CA). *p*-values < 0.05 were considered significant.

## Results

**FIGURE 2:**
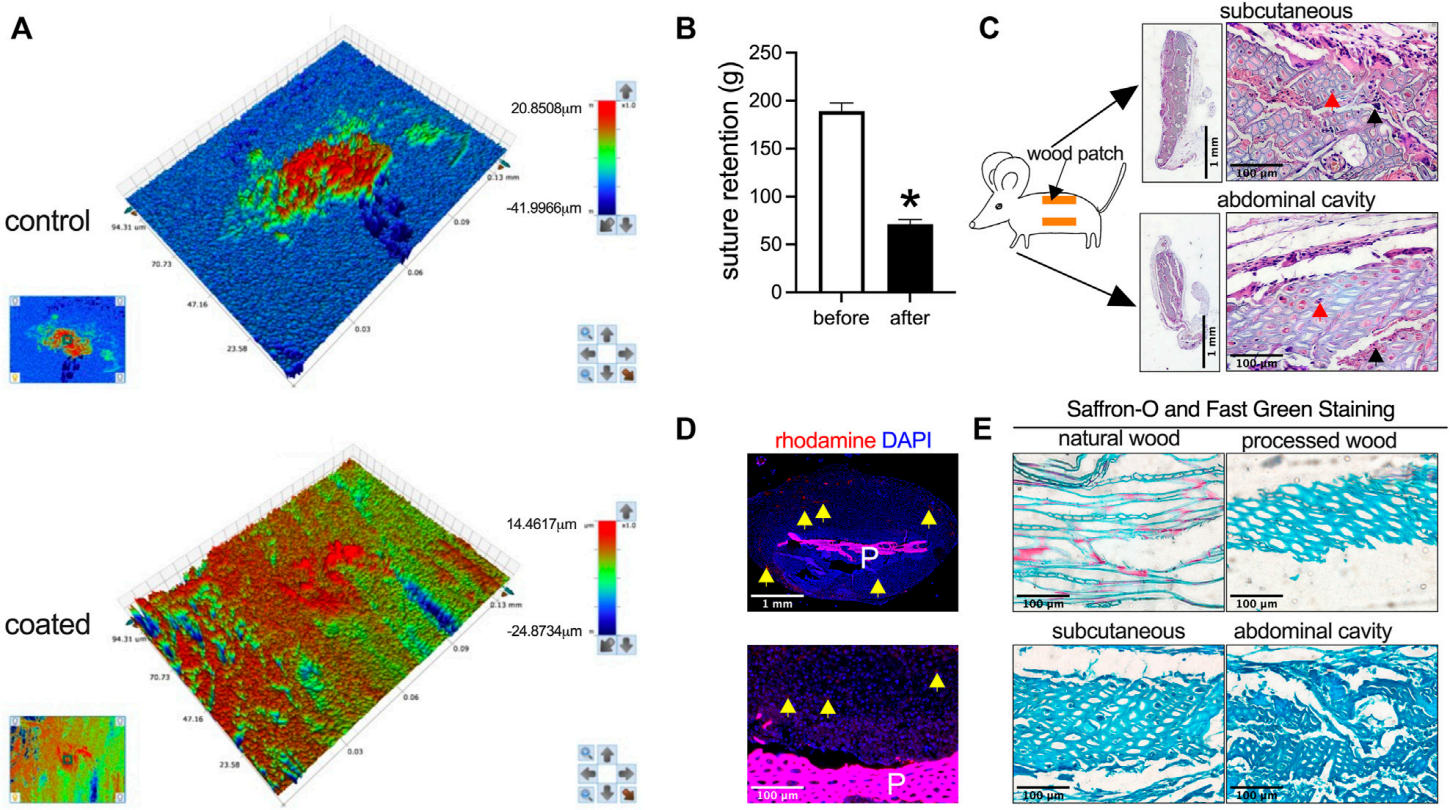
Biocompatibility of wood patches in the rat subcutaneous tissue and the abdominal cavity. **(A)** Representative 3D optical microscopy images of control and HA-coated wood patches (*n* = 3). **(B)** Bar graph showing the suture retention (*p = 0.0003; *t*-test; *n* = 3). **(C)** Low and high-power photographs of hematoxylin and eosin (H&E) staining of the wood patch harvested on day 14 from rat subcutaneous tissue and the abdominal cavity; black arrowhead shows cell infiltration along the interspace between the fibers; red arrowhead shows cell infiltration into the fibers; scale bar, 1 mm or 100 μm; *n* = 3. **(D)** Immunofluorescence showing the rhodamine-conjugated wood patch harvested on day 14 from the rat subcutaneous tissue; upper panel shows low power, and lower panel shows high power; P, patch; scale bar, 1 mm or 100 μm; yellow arrow showing the rhodamine fluorescence; *n* = 3. **(E)** Saffron-O and Fast Green Staining of the natural wood and processed wood, harvested from the rat subcutaneous tissue and abdominal cavity; the red staining of the lignified wood tissue in the natural wood should be noted; scale bar 100 μm; *n* = 3.

The wood used for patch preparation was derived from the trunk rings, and processing yielded soft slices (Figure 1). 3D optical microscopy was used to assess uncoated and HA-coated patches; uncoated and HA-coated patches displayed distinct surface morphologies, with uncoated control patches showing heterogeneity and HA-coated patches showing more uniform morphology (Figure 2A). However, processing reduced material strength as measured by suture retention (Figure 2B). To determine the biocompatibility of these processed wood patches, the patches were implanted in the subcutaneous tissue and abdominal cavity of rats. In both locations the patches became encapsulated; microscopic observation showed cells infiltrated into the wood patches (Figure 2C). Wood patches were also loaded with rhodamine and implanted into the subcutaneous tissue; immunofluorescence showed the rhodamine released from the patch into the surrounding tissue, confirming the successful coating and release of a therapeutic drug from these patches (Figure 2D). Saffron-O and Fast Green Staining showed red staining of the lignified wood tissue in the natural wood, but there was no red-stained lignified wood tissue after processing (Figure 2E), suggesting that the processing was complete; similarly, Saffron-O and Fast Green Staining showed no red-stained lignified wood tissue after implantation into either subcutaneous tissue or abdominal cavity implantation (Figure 2E). These data suggest the biocompatibility of wood-derived patches and their potential utility as a drug delivery system.

**FIGURE 3:**
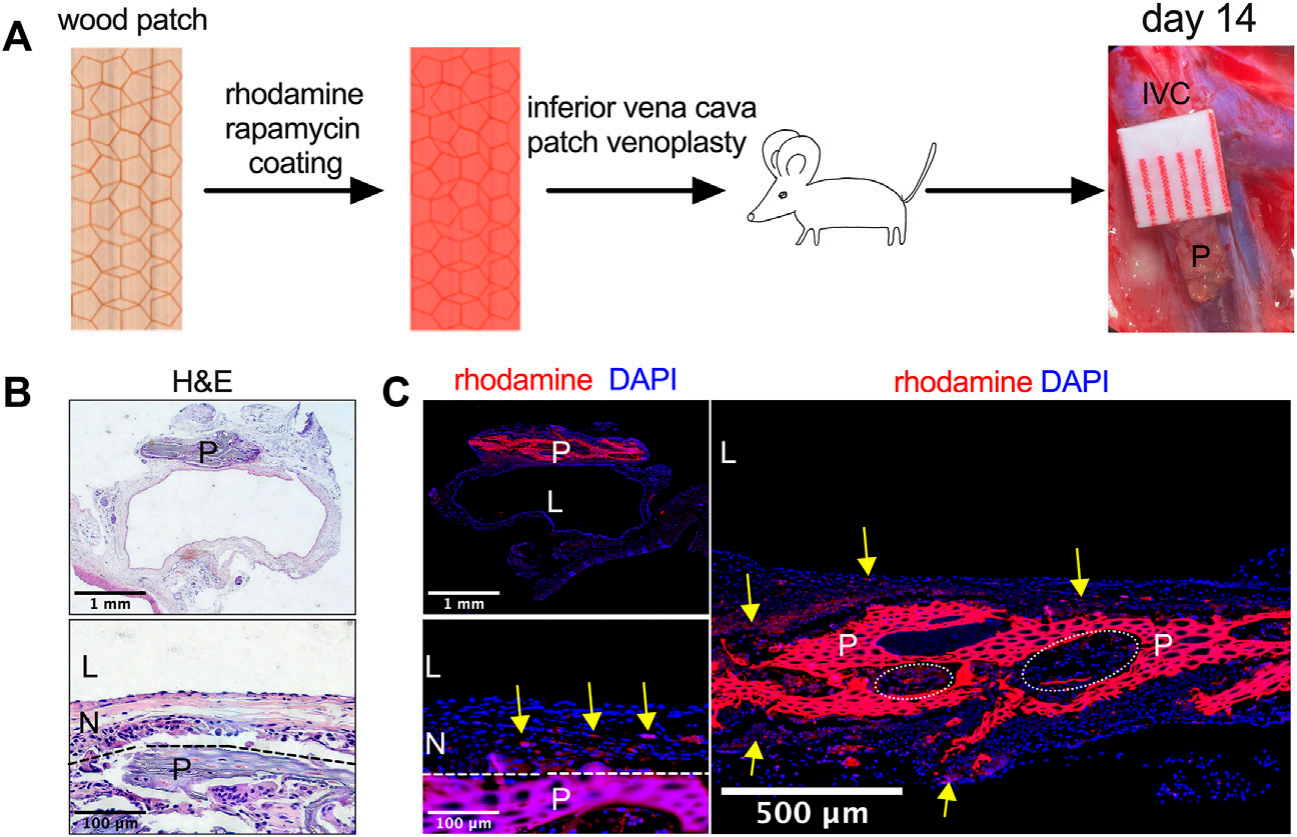
Utility of rhodamine and rapamycin-coated wood patches as a drug delivery scaffold. **(A)** Experimental schema. IVC, inferior vena cava; P, patch. **(B)** Photographs of a patch stained with hematoxylin and eosin (H&E) on day 14; P, patch; L, lumen; N, neointima; *n* = 3; the dashed line shows demarcation of the neointima and the wood patch. **(C)** Immunofluorescence showing the rhodamine-conjugated wood patch harvested on day 14 after patch venoplasty, P, patch; L, lumen; N, neointima; scale bar, 1 mm or 100 μm or 500 μm; yellow arrow shows the rhodamine fluorescence; *n* = 3; cell infiltration into the wood patch denoted by the dashed line circles should be noted.

Since tissue-engineered planted-derived patches show biocompatibility in rats, wood patches were implanted into the IVC of a rat to determine whether wood patches can function as a drug delivery scaffold for vascular applications. Wood patches were coated with rhodamine and rapamycin and then implanted into the rat IVC; patches were incorporated into the native IVC without an intense foreign body reaction (Figure 3A). No patches burst. Hematoxylin and eosin (H&E) staining showed a lack of any mural thrombus formation; there was a well-formed tissue that formed on both the luminal side (‘neointima’) and the abdominal cavity side (‘adventitia’) of the patches; cells migrated and infiltrated into the interspaces of the wood patches (Figure 3B). Immunofluorescence showed rhodamine released from the patches into the surrounding neointima and adventitia tissue (Figure 3C). These data show that wood can be successfully used as a vascular patch and as a novel drug delivery scaffold and also suggest that therapeutic drugs can be successfully released from the patch into the surrounding tissues and detectable for at least 14 days.

**FIGURE 4:**
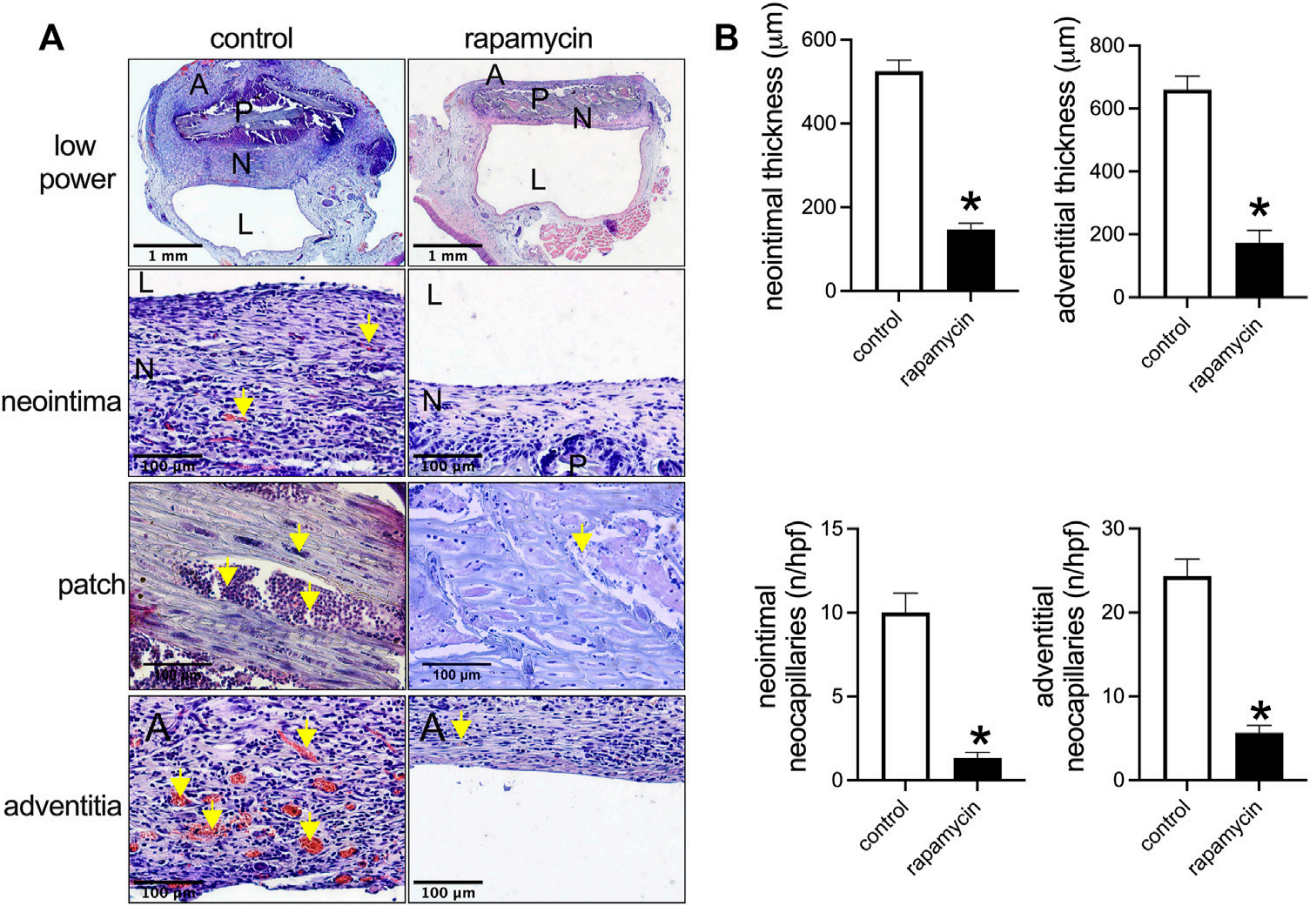
Rapamycin-coated wood patches decrease venous neointimal hyperplasia. **(A)** First row: low power microphotographs of hematoxylin and eosin (H&E) staining showing the control and rapamycin-coated wood patches harvested on day 14; second row: high power showing the neointima; third row: high power showing the patch; fourth row: high power showing the adventitia; P, patch; N, neointima; L, lumen; yellow arrow shows neocapillaries or infiltrated cells; scale bar, 1 mm and 100 μm. **(B)** Bar graphs show neointimal thickness (*p = 0.0001, *t*-test), adventitial thickness, neointimal neocapillaries (*p = 0.0001, *t*-test), and adventitial neocapillaries (*p = 0.0001, *t*-test); n = 3.

To examine the effect of drug delivery, wood patches were loaded with rapamycin. Wood patches without rapamycin (control) showed a thick neointima and adventitia, whereas wood patches coated with rapamycin showed a significantly thinner neointima and adventitia (Figures 4A,4B). In addition, in the neointima and adventitia, there were significantly fewer neocapillaries near patches treated with rapamycin than control patches (Figures 4A,4B). Cells infiltrated into the spaces of the wood patches in both the control and rapamycin-treated patches (Figure 4A).

**FIGURE 5:**
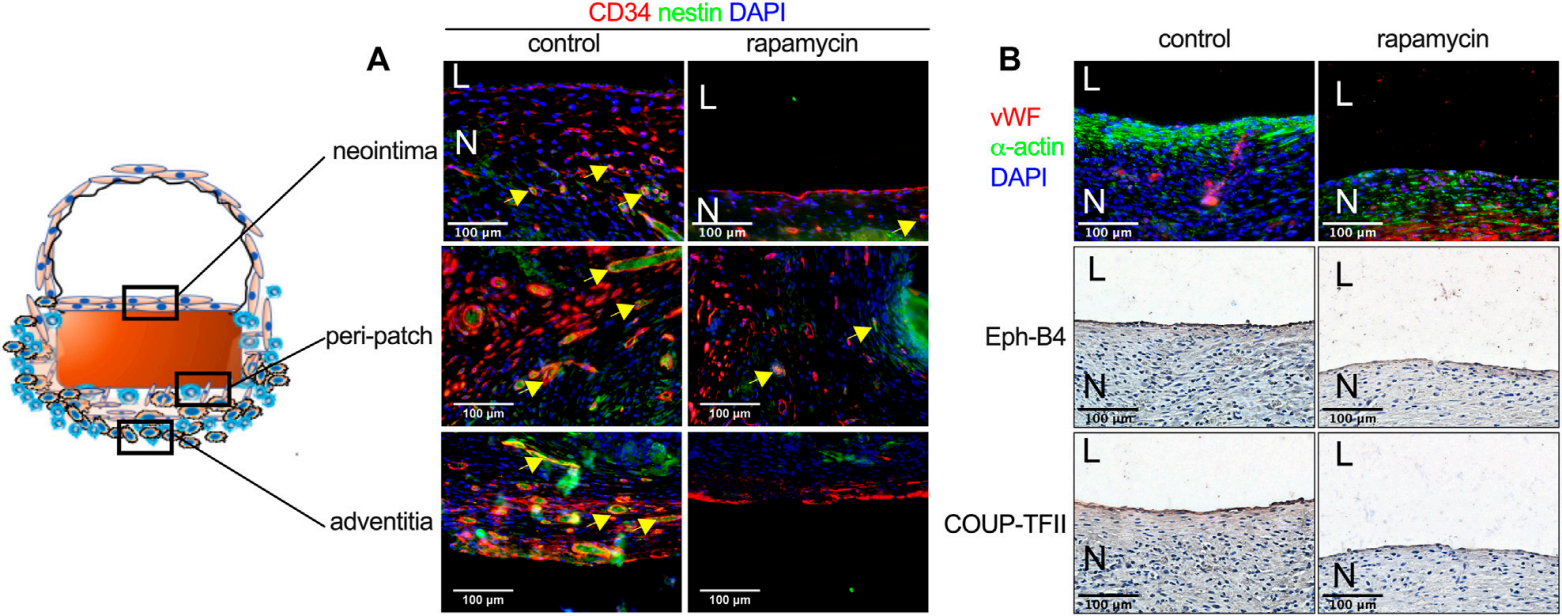
Rapamycin does not influence neointimal endothelial progenitor cell migration or venous identity. **(A)** Immunofluorescence showing CD34 (red), nestin (green), and DAPI (blue) in the neointima, the peri-patch area, and the adventitia yellow arrow showing the positive cells, scale bar, 100 µm; *n* = 3. **(B)** First row: immunofluorescence showing vWF (red), α-actin (green) and DAPI (blue) in the neointima; second and third rows: immunohistochemistry showing Eph-B4 and COUP-TFII, respectively; scale bar, 100 µm *n* = 3.

CD34 and nestin are markers of progenitor cells that participate in neointimal formation during patch healing (Bai et al., 2022b). There were CD34 and nestin-positive cells in the neointima, in the ‘peri-patch’ area (the new tissue adjacent to the patch), and in the adventitia; some cells were both CD34- and nestin dual-positive (Figure 5A). There were fewer CD34 and nestin-positive neocapillaries in the peri-patch area and the adventitia in the rapamycin-treated patches (Figure 5A). Immunofluorescence also showed that there were vWF-positive cells and several layers of α-actin-positive cells in the neointima in both groups (Figure 5B). Eph-B4 and COUP-TFII are markers of venous endothelial cell identity (Bai et al., 2021c); immunohistochemistry showed that the endothelial cells on the neointimal surface were Eph-B4- and COUP-TFII-positive (Figure 5B), consistent with their placement in the IVC.

**FIGURE 6:**
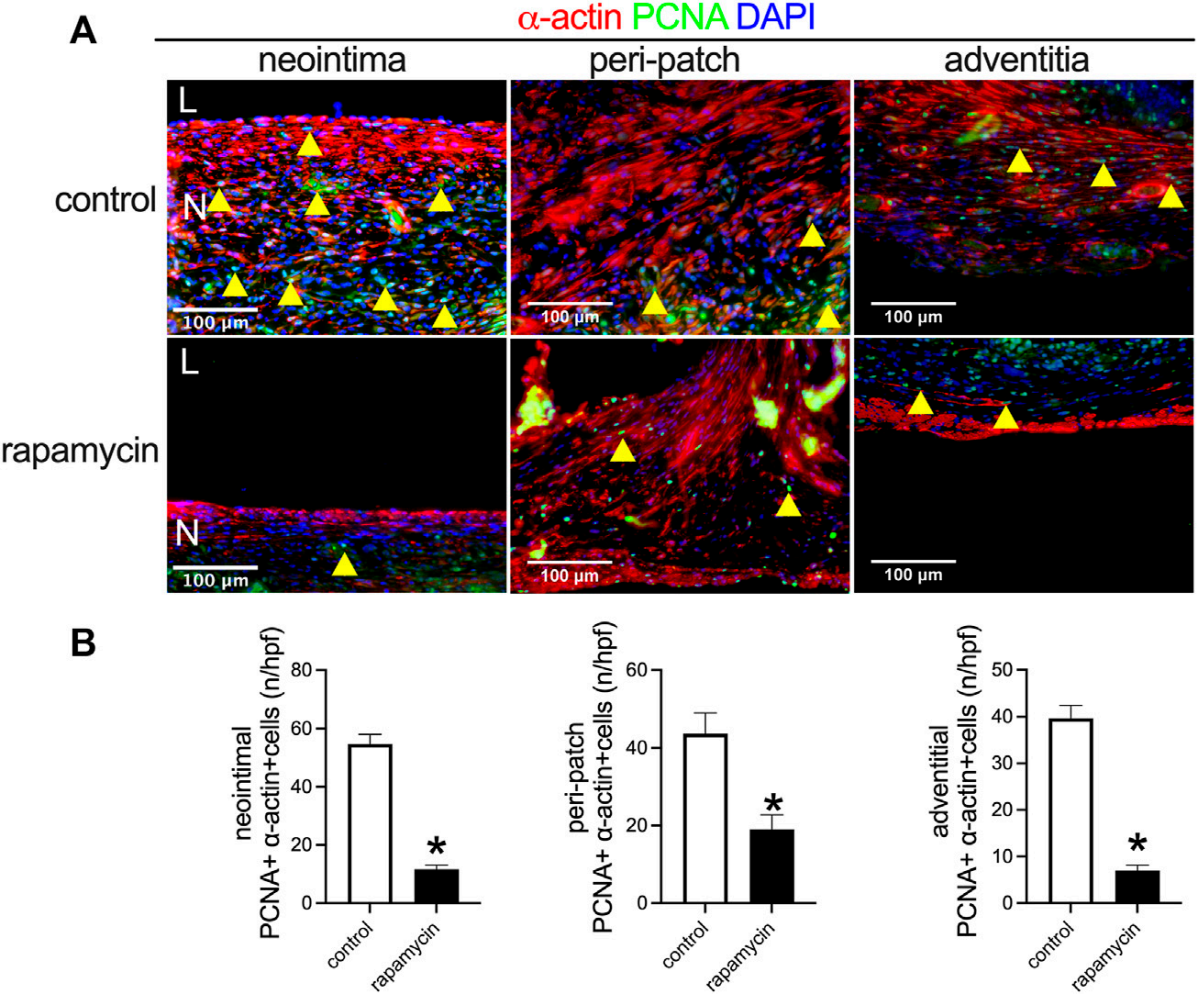
Rapamycin inhibits neointimal smooth muscle cell proliferation. **(A)** Immunofluorescence showing PCNA (green), α-actin (red), and DAPI (blue) in the neointima; scale bar 100 μm; yellow arrowheads show PNCA-positive cells. **(B)** Bar graphs showing quantification of PCNA-positive cells (*, *p* = 0.0003, *t*-test) in the neointima (*p = 0.0073, *t*-test); *n* = 3.

Rapamycin inhibits cell proliferation to reduce neointimal hyperplasia (Bai et al., 2017b; Bai et al., 2021a). In both the control and rapamycin-coated wood patches, PCNA-positive cells were found interspersed throughout the neointima, with some PCNA-positive smooth muscle cells in the peri-patch area and the adventitia (Figure 6A). There were significantly fewer PCNA and α-actin dual-positive cells in the neointima and in the peri-patch area in the rapamycin-treated patches than control patches (Figures 6A,6B). These data show that wood patches can be loaded with rapamycin to effectively decrease neointimal hyperplasia.

**FIGURE 7:**
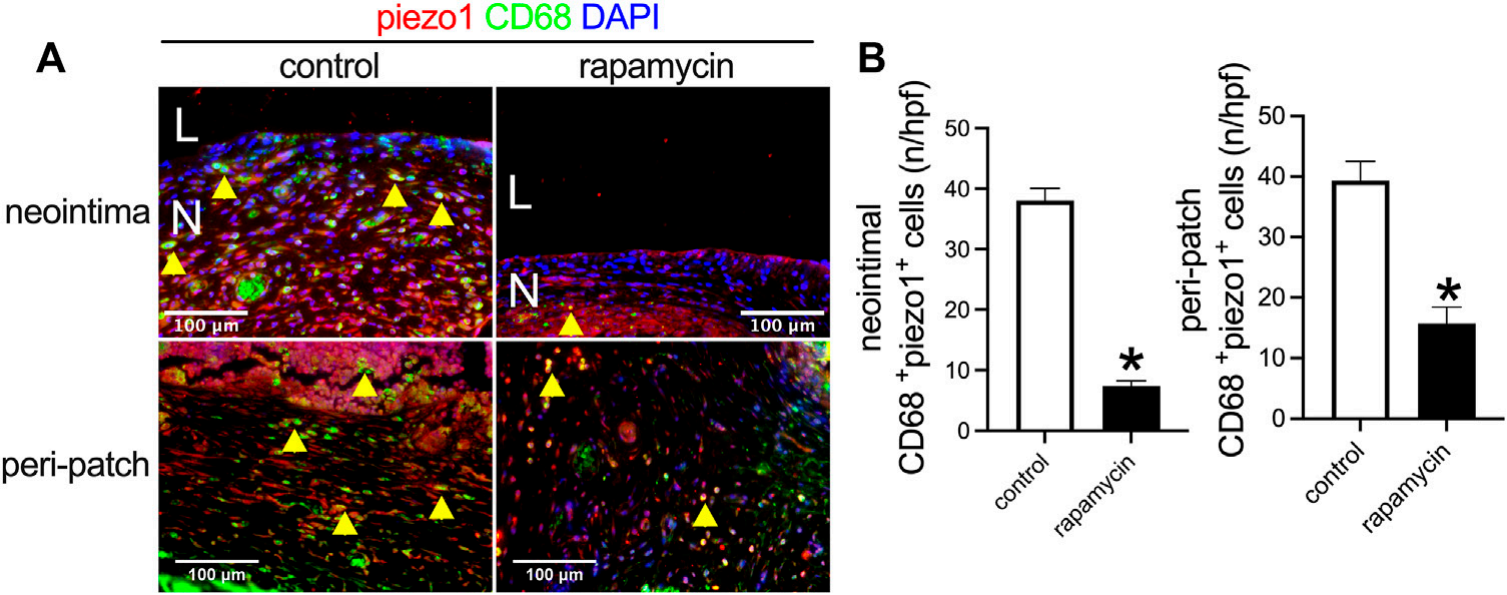
Piezo1 expression after wood patch venoplasty. **(A)** Immunofluorescence showing CD68 (green), piezo1 (red), and DAPI (blue) in the neointima; scale bar 100 μm; yellow arrowheads show CD68 and piezo1 dual-positive cells; N, neointima; L, lumen; *n* = 3. **(B)** Bar graphs showing CD68 and piezo1 dual-positive cells in the neointima (*p = 0.0001, *t*-test) and the adventitia (*p = 0.0018, *t*-test); *n* = 3.

Piezo1 is a mechanosensitive ion channel in mammals that plays a role in the macrophage response to implanted stiff materials (Atcha et al., 2021). Since wood is a stiff material compared to the native IVC, we examined piezo1 expression in the control patches and rapamycin-treated patches. There were significantly fewer piezo1-positive cells in the patches treated with rapamycin than control patches (Figures 7A,7B); some of the macrophages were piezo1-positive. These data show that wooden patches treated to deliver rapamycin have reduced piezo1 expression, suggesting their increased biocompatibility.

## Discussion

We show that wood can be softened to allow successful implantation and be used as a novel vascular patch. In addition, wood patches can also be loaded with rapamycin to inhibit neointimal hyperplasia, suggesting their utility as a novel drug delivery scaffold. These data suggest that plant-based materials can be used as vascular patches, a potential replacement for currently used animal-based materials.

Multiple materials can be used as vascular patches in addition to the currently used ePTFE-, polyester-, vein-, pericardium-, and polycaprolactone-based patches (Bai et al., 2022a; Bai et al., 2020; Bai et al., 2017c; Bai et al., 2017a). Natural scaffolds may be created from diverse sources such as the decellularized fish swim bladder (Bai et al., 2021b), SA/HA hydrogel patches (Wei et al., 2022), biomimetic elastin patches (Bai et al., 2021c), eggshell membrane patches (Sun et al., 2022), and plant-derived patches (Bai et al., 2021a; Xie et al., 2021). These patches showed different compliance and stiffness, reflecting different mechanical properties. Wood patches, at least as processed with the previously published softening protocol, are biocompatible, but reduced strength prevents its use in the high-pressure arterial environment, confining the use of these wood patches to the lower pressure venous environment. Future modifications to the processing steps may allow an increased strength and thus be applied to the arterial environment.

Recently, plant-derived scaffolds have been used in cell culture and show promising applications (Contessi Negrini et al., 2020; Gershlak et al., 2017) and continue to attract attention (Latour and Pelling 2022; Ianovici et al., 2022). Plant-based scaffolds can be used as a vascular patch and a drug delivery system (Xie et al., 2021; Bai et al., 2021a). Here, we extend these data to wood-based patches. Wood is mainly composed of polysaccharides (cellulose, hemicelluloses, and pectins), lignin, and cell wall proteins (Jamet and Dunand 2020). Wood-based scaffolds have some special merits; they have a naturally aligned microchannel structure, and these microchannels can be a microenvironment for cell attachment, migration, and proliferation. Traditional patch materials such as ePTFE do not have these microstructures, and thus, cells rarely infiltrate into them (Kiritani et al., 2020); polyester materials have larger spaces between fibers that allow cell infiltration (Bai et al., 2017a). Pericardial patches support limited cell migration and infiltration into the edge of the patch, but cells do not migrate into the center of the patch that has a tighter microstructure (Bai et al., 2017b). Leaf-based patches do not support cell migration due to the tight structure of the cuticle surface (Bai et al., 2021b). We show that wood-based patches support cell migration throughout the patch, including the patch center, potentially increasing the healing and regeneration of wood patches. However, we did not compare the different potential mechanisms of patch healing among wooden and other patches such as polyester patches (Bai et al., 2017c), pericardial patches (Bai et al., 2017a), decellularized saphenous vein patches (Bai et al., 2020), or biodegraded polycaprolactone and gelatin-fabricated patches (Bai et al., 2022a).

Physical properties of the tissue regulate macrophage behavior, and recent research showed that piezo1 is involved in macrophage sensing of microenvironmental stiffness; macrophages lacking piezo1 show reduced inflammation and enhanced wound healing responses, and stiffness-dependent changes in macrophage function require piezo1 (Atcha et al., 2021). Piezo1 is highly expressed in the neointima and the peri-patch area around wood patches, and reduced neointimal thickness was associated with the reduced piezo1 expression (Figure 7); these data suggest high stiffness of the processed wood patch but may also reflect compliance mismatch between the wood patch and the native IVC. Additional research on the role of piezo1 expression in patch healing should be explored. In addition, research is needed to optimize the processes of wood preparation including the determination of how different protocols alter wood stiffness and strength, as well as the effect of processing on different woods sourced from different tree species; increasing the strength of the wood patch could allow additional applications including use as an arterial patch.

The mammalian target of rapamycin (mTOR) is an atypical protein kinase that controls growth and metabolism in response to nutrients, growth factors, and cellular energy levels; rapamycin is an allosteric inhibitor of mTOR and was approved as an immuno suppressant for human use in 1999 (Benjamin et al., 2011). Rapamycin is recognized and clinically used to inhibit neointimal hyperplasia after vascular interventions since rapamycin inhibits cell proliferation both in the vascular neointima and the adventitia (Bai et al., 2021c). Rapamycin has also been used to prevent neovascularization by downregulation of cyclin D1 in a mouse model of oxygen-induced retinopathy (Jiang et al., 2020), and rapamycin inhibits proliferation and formation of neocapillaries in the peri-patch area after patch implantation (Bai et al., 2021a). We showed that rapamycin was functional when delivered using wood patches (Figure 4), suggesting that wooden patches can serve as an implantable drug delivery system; other drugs may be similarly delivered and require additional investigation. In addition, rapamycin may function *via* a piezo1-mediated mechanism (Figure 7), suggesting that the effects of rapamycin in this model are complex, requiring additional mechanistic experiments.

## Conclusion

Wood-derived scaffolds can be used successfully as vascular patches and a drug delivery system, suggesting the potential application of plant-derived materials in vascular applications. The natural microstructure in the wood patch facilitates cell attachment and migration, as well as therapeutic drug loading and continuous release *in vivo*. Future research is needed to determine the optimal wood source and processing protocols to extend biological applications.

## Data Availability

The original contributions presented in the study are included in the article and Supplementary Material; further inquiries can be directed to the corresponding authors.
